# The First Nationwide Survey and Genetic Analyses of Bardet-Biedl Syndrome in Japan

**DOI:** 10.1371/journal.pone.0136317

**Published:** 2015-09-01

**Authors:** Makito Hirano, Wataru Satake, Kenji Ihara, Ikuya Tsuge, Shuji Kondo, Ken Saida, Hiroyuki Betsui, Kazuhiro Okubo, Hikaru Sakamoto, Shuichi Ueno, Yasushi Ikuno, Ryu Ishihara, Hiromi Iwahashi, Mitsuru Ohishi, Toshiyuki Mano, Toshihide Yamashita, Yutaka Suzuki, Yusaku Nakamura, Susumu Kusunoki, Tatsushi Toda

**Affiliations:** 1 Department of Neurology, Sakai Hospital Kinki University Faculty of Medicine, Sakai, Japan; 2 Department of Neurology, Kinki University Faculty of Medicine, Osakasayama, Japan; 3 Division of Neurology/Molecular Brain Science, Kobe University Graduate School of Medicine, Kobe, Japan; 4 Department of Pediatrics, Kyushu University Graduate School of Medicine, Fukuoka, Japan; 5 Department of Pediatrics, Fujita Health University, Toyoake, Japan; 6 Department of Pediatrics, Tokushima University, Tokushima, Japan; 7 Department of Nephrology, National Center for Child Health and Development, Tokyo, Japan; 8 Department of Pediatrics, Haga Red Cross Hospital, Mooka, Japan; 9 Department of Ophthalmology, Osaka University Graduate School of Medicine, Suita, Japan; 10 Department of Gastrointestinal Oncology, Osaka Medical Center for Cancer and Cardiovascular Diseases, Osaka, Japan; 11 Department of Metabolic Medicine, Osaka University Graduate School of Medicine, Suita, Japan; 12 Department of Geriatric Medicine, Osaka University Graduate School of Medicine, Suita, Japan; 13 Department of Pediatric Neurology, Osaka Medical Center and Research Institute for Maternal and Child Health, Izumi, Japan; 14 Department of Molecular Neuroscience, Osaka University Graduate School of Medicine, Suita, Japan; 15 Department of Computational Biology, Graduate School of Frontier Sciences, The University of Tokyo, Kashiwa, Japan; National Eye Institute, UNITED STATES

## Abstract

Bardet-Biedl syndrome (BBS) is an autosomal recessive disorder characterized by central obesity, mental impairment, rod-cone dystrophy, polydactyly, hypogonadism in males, and renal abnormalities. The causative genes have been identified as *BBS1-19*. In Western countries, this disease is often reported, but remains undiagnosed in many patients until later in life, while only a few patients with no mutations identified have been reported in Japan. We thus conducted the first nationwide survey of BBS in Japan by sending questionnaires to 2,166 clinical departments with board-certified specialists and found 7 patients with clinically definite BBS. We performed exome analyses combined with analyses of mRNA and protein in these patients. We identified 2 novel mutations in the *BBS5* gene (p.R89X and IVS7-27 T>G) in 2 sibling patients. The latter mutation that resided far from the authentic splicing site was associated with skipping of exon 8. We also found 3 previously reported mutations in the *BBS2* (p.R413X and p.R480X) and *BBS7* (p.C243Y) genes in 2 patients. To our knowledge, a nationwide survey of BBS has not been reported in any other country. In addition, this is the first study to identify genetic alterations in Japanese patients with BBS. Our results indicate that BBS in Japan is genetically heterogeneous and at least partly shares genetic features with BBS in other countries.

## Introduction

Bardet-Biedl syndrome (BBS) is an autosomal recessive disorder characterized by central obesity, mental impairment, rod-cone dystrophy, polydactyly, hypogonadism in males, and renal abnormalities. The causative genes have been identified as *BBS1-19*. The prevalence of BBS varies among regions. In Newfoundland, Canada the prevalence is 1:17,500 live births [1]. A similar prevalence has been reported in the Bedouin population of Kuwait (1:13,500) [1, 2]. In contrast, the prevalence is lower in Switzerland (1:160,000) and the United Kingdom (1:125,000) [1, 2]. In Western countries this disease is often reported, but remains undiagnosed in many patients until later in life [1]. In Japan, only a few patients have been reported [3–7], and no mutations have been identified to date. This fact may be attributed to several factors, such as the lack of specialized institutes for BBS that can analyze all the 19 responsible genes in Japan and the presence of racially distinct genetic backgrounds. This study was originally initiated in response to governmental research projects for nationwide surveys of rare diseases, and the research group was subsequently expanded to enable genetic analyses of BBS. We found several patients with clinically definite BBS. Furthermore, exome analyses combined with analyses of mRNA and protein identified the first Japanese patients with genetically definite BBS.

## Materials and Methods

### Patients

A nationwide survey of BBS was conducted in 2010–2011 with the use of two-step questionnaires. The first step was a questionnaire survey inquiring about the number of patients who had central obesity, mental impairment, rod-cone dystrophy, polydactyly, hypogonadism in males, renal abnormalities, and any combinations of these 6 categories of signs and symptoms. It also asked whether potential recessive inheritance (consanguinity and similar diseases in siblings) and *BBS* gene tests were performed. This questionnaire was sent to 2,166 clinical departments with board-certified specialists in neurology (765), child neurology (124), obesity (109), retina and vitreous diseases (115), hepatology (367), nephrology (580), and surgery of the foot (106), the locations of which covered all 47 prefectures in Japan. There appeared to be a few caveats in our nationwide survey: clinical geneticists and pediatric ophthalmologists were not included. In Japan, most clinical geneticists have their own specialties, such as neurology or child neurology. Similarly, most pediatric ophthalmologists also see adults. In addition, ophthalmologists who exclusively evaluate retinal degeneration in children were included in our survey. Because of these conditions in Japan, our survey does not seem to have large caveats.

The second step was to inquire about more detailed clinical/laboratory/genetic information on potential patients who had at least 4 of the 6 categories of features described above or 3 of 6 primary features and 2 secondary features [8]. The secondary features included speech disorder/delay; strabismus/cataracts/astigmatism; brachydactyly/syndactyly; developmental delay; polyuria/polydipsia (nephrogenic diabetes insipidus); ataxia/poor coordination/imbalance; mild spasticity (especially lower limbs); diabetes mellitus; dental crowding/ hypodontia/small roots/high arched palate; left ventricular hypertrophy/congenital heart disease; and hepatic fibrosis. If a patient was suspected to have BBS, genetic analyses were performed after obtaining written informed consent. This study was approved by the Institutional Review Boards of Kinki University and of Kobe University.

### Genetic analyses

For genetic analysis, a DNA chip study was performed at Asper Biotech Ltd. (Tartu, Estonia), after obtaining informed consent from the patient and his or her parents or guardians. The DNA chip (version 5) analysis covered 131 pathological mutations from 13 genes known to cause BBS (*BBS1-13*). We then performed exome sequencing in these patients. Briefly, genomic DNA was captured using a SureSelect Human All Exon v4 kit (Agilent Technologies, Santa Clara, CA) and sequenced using a high-throughput sequencing platform (HiSeq2000, Illumina, San Diego, CA). Reads were aligned to the human genome reference sequence (hg19) with the Burrows-Wheeler Aligner program, and single-nucleotide variants and small insertions, deletions, or both were identified using the Genome Analysis Toolkit (GATK). We first focused on the known *BBS* genes and then extended the search to other possible genes in which mutations commonly resided. All mutations found in this study were confirmed by PCR-direct sequencing, performed using the Sanger-based method.

### RNA analyses

Total RNA from lymphocytes, lymphoblasts, or fibroblasts of control, Patients 1 and 2, and their parents was extracted and reverse transcribed with oligo dT primers. The following primers were used: BBS5mRNAF 5’-ACGCAGCTAGGCCTGCACGGCTGT-3’ and BBS5mRNAR 5’-TATTCCATGACTTATGGCAGGTGAC-3’ for amplification of full-length transcripts; BBS5mRNA455F TAAGACTGTTGCCACAAGAACATG (on exon 6) and BBS5mRNA542R AAAAAGGTTCCTAAATTGCCCTGA (on exons 6 and 7) for sequencing; and BBS5mRNA681R CTGCTGAGAGCTTTCTATGACAAG (on exon 8), BBS5mRNA179F ACTCTTTGGCATTATCAAGAGTCA (on exon 3), and BBS5mRNA740R ACTGATTCTTGTAGTTTTTCCACAG (on exon 9) for analyses of splicing.

### Immunoblot analyses

Whole cell lysates from fibroblasts of control and Patients 1 and 2 were immunoblotted with the antibody against full-length human BBS5 (Proteintech Group, Inc., Chicago, IL, USA) and that against glyceraldehyde 3-phosphate dehydrogenase (GAPDH, Abcam, Cambridge, MA, USA).

## Results

### Results of the nationwide survey

Completed questionnaires were received from 561 departments or hospitals (response rate, 26%). Fifteen of these departments or hospitals had 38 patients with suspected BBS who had at least 4 of the 6 primary features or 3 of the 6 primary features with two secondary features. Follow-up questionnaires were sent to these centers, and more detailed information was available for 9 patients, 7 of whom had clinically definite BBS. Genetic tests were performed in these 7 patients. The results showed that 4 patients had pathological mutations: 2 had novel mutations in the *BBS5* gene, and the other 2 patients had known mutations in the *BBS2* and *BBS7* genes, respectively, as described below in detail. The clinical and genetic characteristics of the patients with genetically definite BBS are summarized in Table 1.

**Table 1:** Clinical and genetic information on Japanese patients with Bardet-Biedl syndrome. ST, strabismus; DC, dental crowding; CA, cardiac anomaly.

Patient #	1	2	3	4
Age	20	16	6	2
Sex	M	M	F	M
Consanguinity	-	-	-	-
Mental impairment	+	+	+	+
Rod-cone dystrophy	+	+	+	?
Obesity	+	+	+	+
Hypogonadism (males)	+	+		+
Polydactyly	-	+	+	+
Renal fibrosis (anomaly)	-	-	+*	-
Hepatic fibrosis	+	-	-	-
Height (cm)	159	166	115	79
Weight (kg)	75	99	32.6	16
BMI (kg/m^2^)	29.7	35.9	24.7	25.6
Note			ST, DC, CA	epilepsy
Causative gene	*BBS5*	*BBS5*	*BBS7*	*BBS2*

*, left mild pyelectasis; BMI, body-mass index.

### Results of genetic analyses and immunoblot analyses

The results of microarray studies showed no pathological point mutations in the *BBS1-13* genes, probably because microarray analysis only covers about one third of currently known causative mutations for BBS. Our exome analyses, however, revealed that 4 patients had pathological mutations in known *BBS* genes. The 3 other patients in whom a mutation was not found fulfilled the clinical criteria for BBS and could not be clinically differentiated from the other patients. Other ciliopathies had not been diagnosed by their physicians. Our exome analyses in this three patients detected no variants of unknown significance in the *BBS* genes identified. Thus far, we have not found any new genes in which mutations commonly resided. Because the estimated frequency of mutation-negative BBS is about 20% in Western countries according to www.genetests.org, we speculate that yet to be determined causative genes exist in Japan.

**Fig 1:**
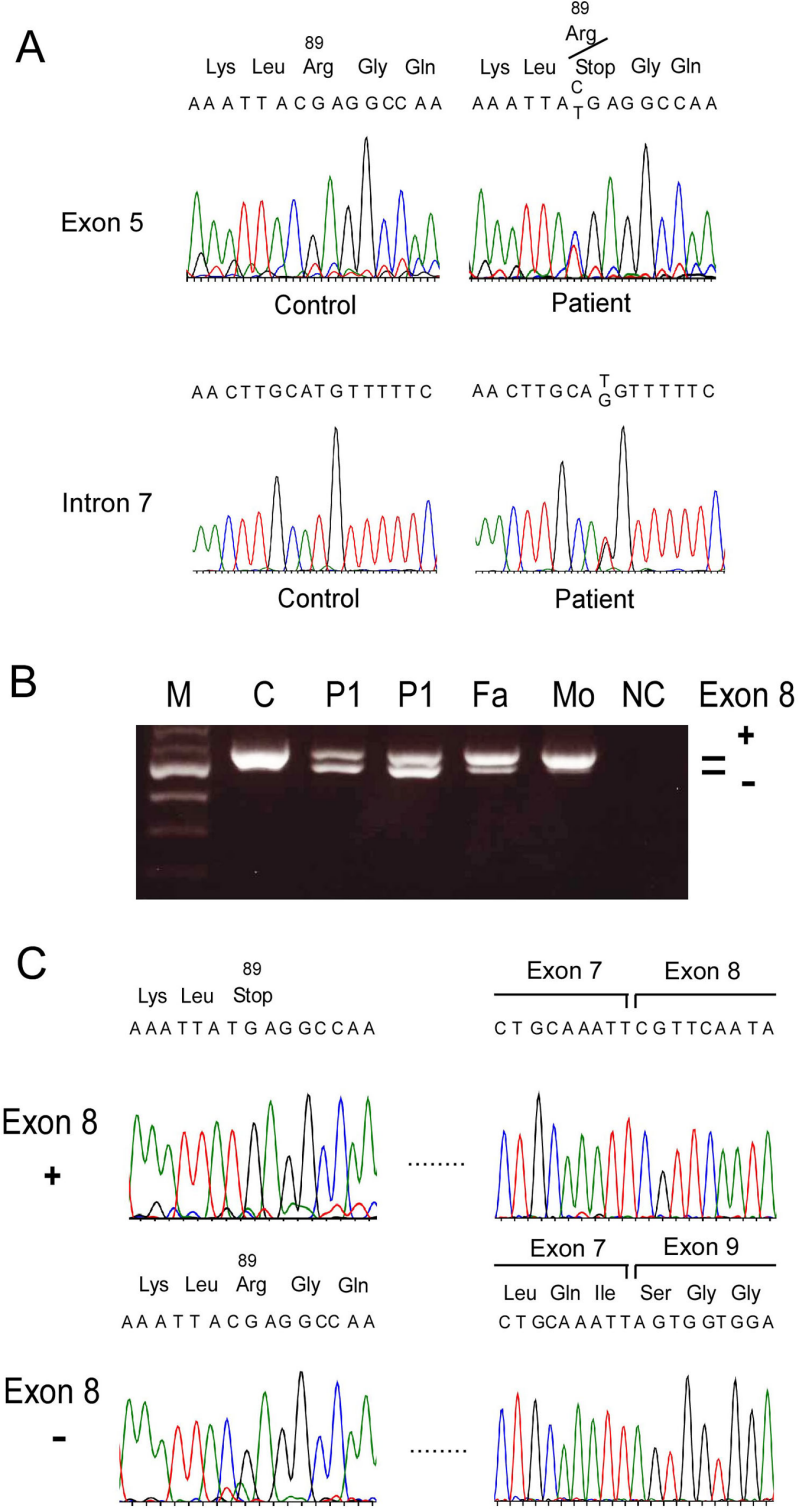
Novel mutations in Patients 1 (P1) and 2 (P2). (A) Genomic DNA sequencing of exon 5 in the *BBS5* gene showed a C>T transition at the codon 89, resulting in arginine to stop (p.R89X). (B) RT-PCR revealed that an extra band with a shorter fragment in P1, P2, and their father (Fa), but not in normal control (C) or their mother (Mo). NC indicates no cDNA contained. (C) Sequencing of RT-PCR fragments showed that the shorter fragment lacked exon 8 with normal sequences in exon 5, while the normal-size fragment included exon 8, but had p.R89X mutation in exon 5. (D) Genomic DNA sequencing in exon 8 and surrounding introns in the *BBS5* gene showed IVS7-27 T>G mutation in the patients.

Exome analyses of 2 siblings, Patients 1 and 2, revealed that they were heterozygous for a novel mutation, p.R89X (c.C265T), in the *BBS5* gene (Fig 1A). No apparently pathological mutation was additionally detected in this gene, although BBS is thought to be an autosomal recessive disease usually associated with alterations in both alleles. We then examined *BBS5* mRNA to find a whole-exon deletion that exome analyses could not detect. Our RT-PCR analyses of fibroblasts and lymphocytes from patients, their parents, and a control showed that the patients and their father had a shorter transcript in addition to an apparently normal-size transcript, while the control and the mother had an apparently single normal-size transcript (Fig 1B). The shorter transcript lacked exon 8 (Fig 1C). Unexpectedly, sequencing of exon 8 and conjunctive introns 7 and 8 (more than 100 bases upstream and downstream of exon 8) in the genomic DNA revealed an only heterozygous IVS7-27 T>G mutation (Fig 1D), suggesting that this mutation is attributed to skipping of exon 8. DNA analyses of the patients’ parents revealed that the father was heterozygous for only IVS7-27 T>G mutation, consistent with the result that the father had the transcript lacking exon 8, and the mother was heterozygous for only p.R89X mutation (not shown). The results of long-range RT-PCR of the patients’ DNA showed that the apparently normal-size transcript exclusively contained p.R89X mutation, while the transcript lacking exon 8 did not contain this nonsense mutation, indicating that the patients had only mutant transcripts encoding truncated proteins. These mutations were not present in our in-house 300 controls or in the 1000 genome database.

**Fig 2:**
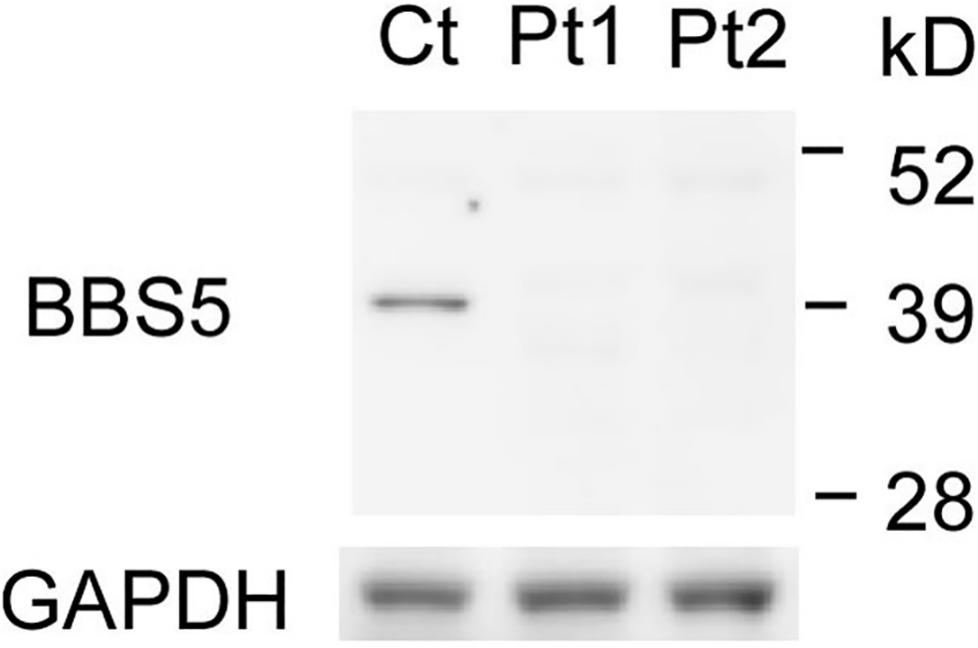
Protein analyses of BBS5. The immunoblot showed that Patients 1 (P1) and 2 (P2) had no full-length BBS5 protein, while control (C) had it. Glyceraldehyde 3-phosphate dehydrogenase (GAPDH) was used as loading control.

Immunoblot of proteins from fibroblasts of control and patients showed no full-length BBS5 protein in 2 patients while a control subject had a single full-length 39 kD protein, consistent with the data from the supplier (Fig 2, http://www.ptglab.com/Products/BBS5-Antibody-14569-1-AP.htm). The in-frame transcript lacking exon 8 was supposed to encode a 36 kD protein, slightly smaller than the full-length protein; however, such a protein was not detected in our immunoblot analyses with the antibody raised against a full-length BBS5 protein.

**Fig 3:**
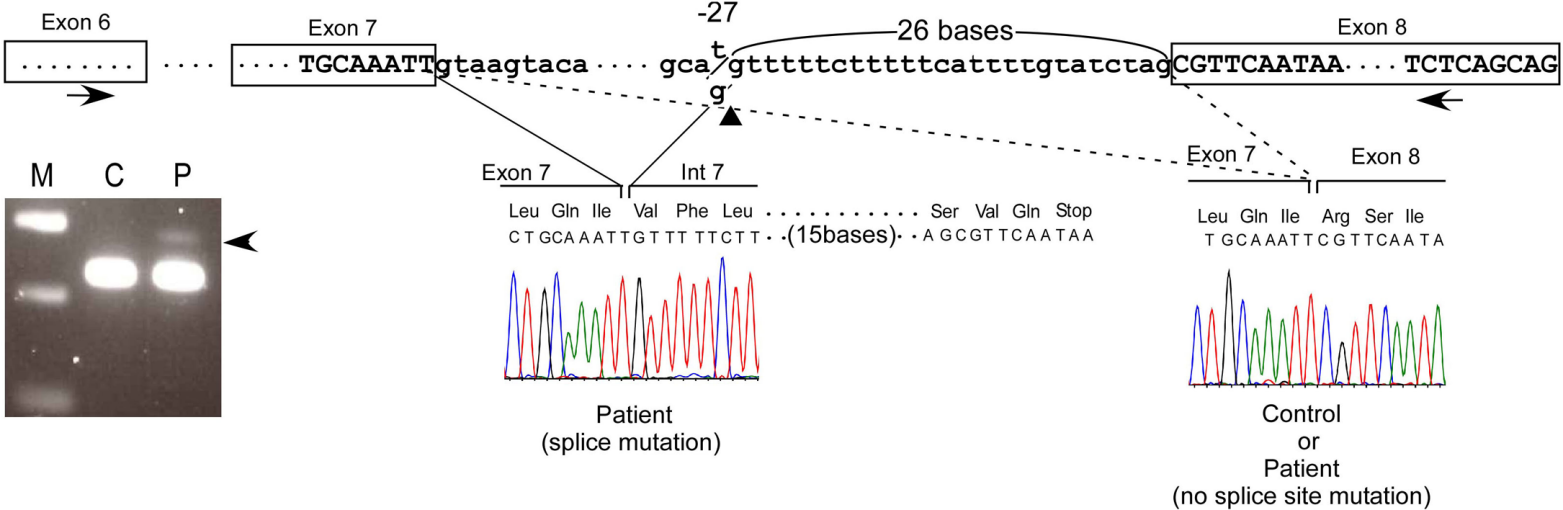
A detection of minor transcript using a new 3’-splice site. In the upper panel, a sensitive RT-PCR spanning a short range (exon 6 to 8) and using primers BBS5mRNA455F and BBS5mRNA681R, showed an extra longer band in addition to a band with expected size in the patient with BBS5 mutation. Sequencing of the minor, longer band disclosed a transcript with 26 extra bases after exon 7, resulting in production of a nonsense codon located 11 aberrant residues after exon 7.

A sensitive RT-PCR assay with short-range amplification by primers BBS5mRNA455F and BBS5mRNA681R, but not with long-range amplification covering a full-length transcript (not shown), detected a very small amount of a transcript with 26 extra bases only in the patients, in addition to a large amount of a normal-size transcript (Fig 3). This aberrant transcript was an out-of-frame transcript with a stop codon after 11 aberrant amino acid residues.

Patient 3 was homozygous for p.C243Y (c.G728A) in the *BBS7* gene (S1A Fig). The parents were heterozygous for the mutation.

Patient 4 was compound heterozygous for p.R413X (c.C1237T) and p.R480X (c.C1438T) in the *BBS2* gene (S1B Fig). His father was heterozygous for p.R413X mutation, and his mother was heterozygous for p.R480X mutation (not shown).

We could not find apparently pathological homozygous or compound heterozygous mutations in the other patients, but could not exclude deletions of the whole exon or mutations in the promoter, deep introns, or other regulatory sequences in the causative genes.

### Clinical information on mutation-positive patients

Patient 1 (p.R89X mutation and IVS7-27 T>G in the *BBS5* gene), a 20-year-old man, had mental retardation (MiniMental State Examination 23; normal >24), rod-cone dystrophy, central obesity (height 158 cm, body weight 63 kg, body-mass index 25.2), and hypogonadism since the age of 5 years. He did not have polydactyly. Esophageal, gastric, and rectal varices developed at the age of 5 years, with fibrosis of the liver. The waist circumference was 83.5 cm at the age of 20 years. The blood pressure was 131/85 mmHg, and the heart rate was 61 beats/min. He had normal heart sounds with clear breath sounds. The serum creatinine level was normal. Ultrasonography showed no anomalies in the kidneys at the age of 20 years. The non-consanguineous parents of Patients 1 and 2 were apparently healthy.

Patient 2 (the younger brother of Patient 1) was a 16-year-old boy who had polydactyly, mental retardation, central obesity (height 165 cm, body weight 93 kg, body-mass index 34.2), and hypogonadism. The waist circumference was 107 cm. The blood pressure was 128/61 mmHg, and the heart rate was 77 beats/min. He had normal heart sounds with clear breath sounds. The serum creatinine level was normal. Ultrasonography showed no anomalies in the kidneys. He did not have hepatic fibrosis.

Patient 3 (homozygous for p.C243Y in the *BBS7* gene), a 6-year-old-girl, had non- consanguineous parents with no similar disease in the family. She had a previous history of bilateral surgical excision of the extra toes associated with polydactyly. She was obese at the age of 1 year. Rod-cone dystrophy became apparent at 3 years of age. Mental impairment was apparent, as the IQ was 44 at the age of 6 years. Although the serum creatinine and BUN levels were normal, the left renal pelvis was mildly enlarged at 6 years of age. No hepatic abnormality was evident.

Patient 4 (compound heterozygous for p.R413X and p.R480X in the *BBS2* gene), a 2-year-old boy, had a Japanese father and a Filipino mother. He had a birth weight of 3,170 g and seizures at the age of 1.75 years. He became obese at the age of 1 year (height 79 cm and body weight 16 kg) and had polydactyly and a small penis. MRI showed enlargement of the lateral ventricles. His mental and verbal development was delayed. No renal anomaly was evident.

## Discussion

This first nationwide survey in Japan revealed that only 7 patients had clinically definite BBS. Four of these patients were the first Japanese patients with genetically definite BBS. Notably, 3 different causative genes for BBS were detected, although the Japanese population is thought to be relatively homogenous genetically. In addition, the 3 mutations detected have been reported in different countries far from Japan. These results suggest that BBS is genetically heterogeneous in Japan and that the genetic backgrounds of patients with BBS in Japan are at least partly consistent with those of patients with BBS in other countries. This is important clinically, since future treatments developed in other countries are likely to be applicable in Japan, and vice versa. In contrast to the usefulness of the exome analyses, the microarray analysis was not as good as a first-line test in this population.

Our analyses of mRNA and protein in Patients 1 and 2 revealed that the novel splice site mutation IVS7-27 T>G seemed to affect splicing of the *BBS5* gene. Together with p.R89X mutation, this mutation may also encode a protein smaller than wild-type protein, which is partly consistent with the results of the immunoblot analyses showing no full-length BBS5 protein in the patients. In addition, the protein encoded by the in-frame transcript lacking exon 8 was undetectable, possibly because a small protein without the middle region may be unstable.

How IVS7-27 T>G mutation affects splicing remains unknown, but a previous study of other genes demonstrated that a newly created AG sequence upstream of the authentic (original) 3’-splice site suppressed the splicing activity, but sometimes did not suffice for exon ligations [9]. For example, in the *FBN2* gene IVS28-15 A>G mutation (AA to AG) suppressed splicing at the authentic site, but did not function as a novel splice site resulting in skipping of the downstream exon 29 [9].

An alternative mechanism may be that the IVS7-27 T>G mutation in the *BBS5* gene creates a cryptic splice site located 26 bases upstream of the original splice site, since the degree of match to the consensus 3’-splice site sequence for the new sequence (74.8%) was almost equivalent to that for the authentic splice site sequence (77.1%) [10]. However, when the new splice site incorporated 26 extra bases into mRNA, it produced an out-of-frame transcript with a stop codon after 11 aberrant amino acid residues. This might have caused nonsense-associated altered splicing (NAS), a mechanism that occurs in a translational-frame-dependent manner, preferentially producing an in-frame transcript instead of an out-of-frame transcript with a premature stop codon [9]. Our findings suggest that NAS may produce an in-frame transcript lacking exon 8. To support our prediction, a sensitive RT-PCR with short-range amplification detected a very small amount of an out-of-frame transcript only in the patient, suggesting that the new splice site created by the IVS7-27 T>G mutation has activity to ligate exons. However, such a transcript became undetectable on a long-range RT-PCR and was replaced by a NAS-related in-frame transcript. One could claim that the other mutation in exon 5, p.R89X, did not cause NAS because the transcript lacking exon 5 is an out-of-frame transcript.

Patient 3 had a homozygous missense mutation that was recently reported in a North American patient with Leber’s congenital amaurosis or juvenile retinitis pigmentosa [11]. Although the complete medical condition is unclear, the patient was a 28-year-old man who had ophthalmologic involvement since the age of 16 years, with no involvement of the central nervous system or kidney or urinary systems, suggesting that the diagnosis of BBS was not established. In contrast, our patient already had typical features of BBS at the age of 6 years. Although ophthalmologic involvement is one of the cardinal features of BBS, it usually develops and progresses slowly; the mean age at onset is 8.5 years of age [8]. Nonetheless, the onset age of ophthalmologic involvement varies considerably (1–32 years old), and thus the reported patient with Leber’s congenital amaurosis might present with full clinical features of BBS subsequently. Why the same mutation causes varied phenotypes remains to be elucidated, but one can speculate that patients have an additional mutation in other *BBS* genes that modify the disease phenotype [12, 13]. Unfortunately, no additional mutation in known causative genes was found in patient 3 to date (data not shown). Such phenotypic differences might thus be attributed to yet unknown genes.

Patient 4 had compound heterozygous mutations, p.R413X and p.R480X. The same combination of mutations was seen in a Northern European family with BBS [14]. The family’s phenotype included obesity, retinitis pigmentosa, polydactyly, motor delay, and speech delay, but not hypogonadism or renal anomaly. Similarly, our patient did not have renal anomaly. These findings indicate that patients with p.R413X and p.R480X mutations had similar phenotypes, despite other genetic or country-specific differences.

Our results suggest that the absence of genetic alterations reported previously in Japanese patients with BBS is attributed to the lack of specialized institutes that can analyze all causative genes for BBS in Japan. However, the presence of Japanese patients with genetically indefinite BBS might suggest that the genetic backgrounds of the Japanese population partially differ from those of populations in Western countries. Of course, because exome analyses apparently had disadvantages for the detection of whole-exon deletions or splice mutations such as those found in our study, our analyses might have failed to identify certain pathological mutations. Future studies should therefore include such more extensive methods for mutation detection and analysis.

In summary, our study provides evidence that the genetic backgrounds of patients with BBS in Japan are at least partly consistent with those of patients with BBS in other countries. Our result might facilitate the enrollment of Japanese patients in international clinical activities, such as the Clinical Registry Investigating Bardet-Biedl Syndrome (CRIBBS) in the United States. CRIBBS gathers comprehensive health information from patients in a single repository, which will be used to comprehend the complex features of BBS and serve as a platform for researchers to develop effective treatments in the future. Further analyses in Japan as in other countries may be necessary to fully clarify the genetic alterations of BBS.

## Supporting Information

S1 Fig Reported mutations in Patients 3 (A) and 4 (B). (A) Genomic DNA sequencing of exon 8 in the *BBS7* gene showed a G>A transition at codon 243, resulting in a cysteine-to-tyrosine substitution (p.C243Y). The parents were heterozygous and Patient 3 was homozygous for this mutation. (B) Genomic DNA sequencing of exon 11 in the *BBS2* gene showed a C>T transition at codon 413, resulting in an arginine to stop codon mutation (p.R413X). Exon 12 in the *BBS2* gene had a C>T transition at codon 480, resulting in arginine to stop codon mutation (p.R480X). Patient 4 was compound heterozygous for these mutations.
(TIF)
