# Supplementary figures and images for: The First Nationwide Survey and Genetic Analyses of Bardet-Biedl Syndrome in Japan
Source: PLoS One. 2015 Sep 1;10(9):e0136317. doi: 10.1371/journal.pone.0136317 (PMC4556711; doi:10.1371/journal.pone.0136317)

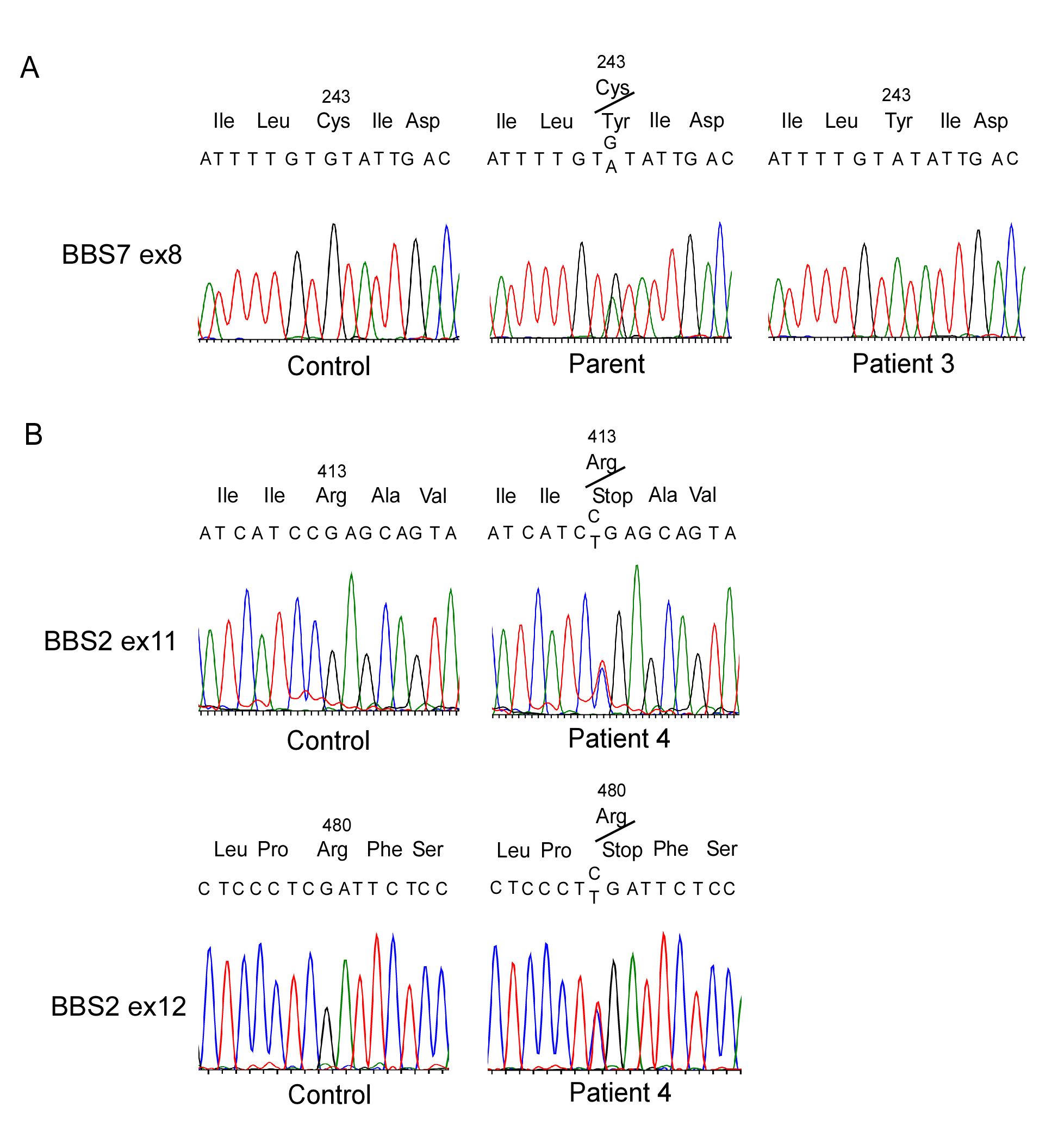

Supplement: S1 Fig — (A) Genomic DNA sequencing of exon 8 in the BBS7 gene showed a G>A transition at codon 243, resulting in a cysteine-to-tyrosine substitution (p.C243Y). The parents were heterozygous and Patient 3 was homozygous for this mutation. (B) Genomic DNA sequencing of exon 11 in the BBS2 gene showed a C>T transition at codon 413, resulting in an arginine to stop codon mutation (p.R413X). Exon 12 in the BBS2 gene had a C>T transition at codon 480, resulting in arginine to stop codon mutation (p.R480X). Patient 4 was compound heterozygous for these mutations. (TIF) [file pone.0136317.s001.tif]
